# Modification of Arabinogalactan Isolated from *Larix sibirica Ledeb.* into Sulfated Derivatives with the Controlled Molecular Weights

**DOI:** 10.3390/molecules26175364

**Published:** 2021-09-03

**Authors:** Yuriy N. Malyar, Natalia Yu. Vasilyeva, Aleksandr S. Kazachenko, Valentina S. Borovkova, Andrei M. Skripnikov, Angelina V. Miroshnikova, Dmitriy V. Zimonin, Vladislav A. Ionin, Anna S. Kazachenko, Noureddine Issaoui

**Affiliations:** 1 School of Non-Ferrous Metals and Material Science, Siberian Federal University, Pr. Svobodny, 79, 660041 Krasnoyarsk, Russia; vasilyeva.nata@mail.ru (N.Y.V.); leo_lion_leo@mail.ru (A.S.K.); bing0015@mail.ru (V.S.B.); and-skripnikov@yandex.ru (A.M.S.); miroshnikova35@gmail.com (A.V.M.); zimonind89@mail.ru (D.V.Z.); sl79490@yandex.ru (V.A.I.); kaalla@list.ru (A.S.K.); 2 Institute of Chemistry and Chemical Technology, Krasnoyarsk Science Center, Siberian Branch, Russian Academy of Sciences, Akademgorodok, 50/24, 660036 Krasnoyarsk, Russia; 3 Laboratory of Quantum and Statistical Physics (LR18ES18), Faculty of Sciences, University of Monastir, Monastir 5079, Tunisia; issaoui_noureddine@yahoo.fr

**Keywords:** arabinogalactan, *Larix sibirica Ledeb.*, sulfated arabinogalactan, molecular weight distribution, gel permeation chromatography, optimization, sulfation

## Abstract

The process of sulfation of arabinogalactan—a natural polysaccharide from *Larix sibirica Ledeb.—*with sulfamic acid in 1,4-dioxane using different activators has been studied for the first time. The dynamics of the molecular weight of sulfated arabinogalactan upon variation in the temperature and time of sulfation of arabinogalactan with sulfamic acid in 1,4-dioxane has been investigated. It has been found that, as the sulfation time increases from 10 to 90 min, the molecular weights of the reaction products grow due to the introduction of sulfate groups without significant destruction of the initial polymer and sulfation products. Sulfation at 95 °C for 20 min yields the products with a higher molecular weight than in the case of sulfation at 85 °C, which is related to an increase in the sulfation rate; however, during the further process occurring under these conditions, sulfation is accompanied by the destruction and the molecular weight of the sulfated polymer decreases. The numerical optimization of arabinogalactan sulfation process has been performed. It has been shown that the optimal parameters for obtaining a product with a high sulfur content are a sulfamic acid amount of 20 mmol per 1 g of arabinogalactan, a process temperature of 85 °C, and a process time of 2.5 h.

## 1. Introduction

The anticoagulant drug most widely used in modern medical practice is heparin, a natural hexosaminoglycan of animal origin. Heparin is used to improve the hemocompatibility of the surface of medical devices [1,2,3]. This natural sulphated linear glucosaminoglycan is produced by the mast cells of some animal tissues Unfortunately, the use of heparin, as well as other modern anticoagulants, can be accompanied by collateral damages, including thrombocytopenia and bleeding, regardless of the mechanism of the anticoagulant effect. A promising alternative to heparin is the sulfated derivatives of plant polysaccharides [2,4,5,6,7,8,9,10,11,12,13,14,15,16,17,18,19,20,21]. Various sulphated polysaccharides are known as heparinoids of both natural and synthetic origins have distinctive anticoagulant properties [2,4,5,6,7,8,9,10,11,12,13,14,15,16,17,18,19,20,21]. Owing to their structural diversity and high negative charge, these biopolymers can bind to many proteins, including receptors, thereby exhibiting a wide range of biological activities.

A promising and available substance alternative to heparin is sulfated arabinogalactan [13,14,15,22,23,24,25,26], a product of chemical modification of the plant polysaccharide arabinogalactan [27,28,29,30]. A significant advantage of arabinogalactan [27,28,29,30] over other biologically active polysaccharides is availability, since a reliable source of its industrial production is larch wood, which is one of the most widespread conifers [28]. Depending on the species, larch contains from 10% to 35% of this valuable polysaccharide [28], which is indicative of the huge actual reserves of the raw material and prospects for mass production of the new arabinogalactan-based bioactive agent [27,28]. On the territory of Siberia and the Far East grow Siberian larch (*L. sibirica Ledeb*), Gmelina (*L. gnielinii Rupr.*), and Cajandera (*L. cajanderi Mayr.*) [28]. The quantitative monosaccharide composition and molecular weight of arabinogalactan vary not only depending on the type of larch, but also within the same species [31]. For galactan-containing polysaccharides, the most important characteristics are the size of the galactan core, the structure of the side chains, as well as the molecular weight and the ability to form intermolecular associates [27,28]. The ratio of galactose and arabinose fragments in AG fractions from western larch wood increases from 2.33:1 to 6.99:1 with an increase in molecular weight from 3 to 79 kDa [27].

**Figure 1:**
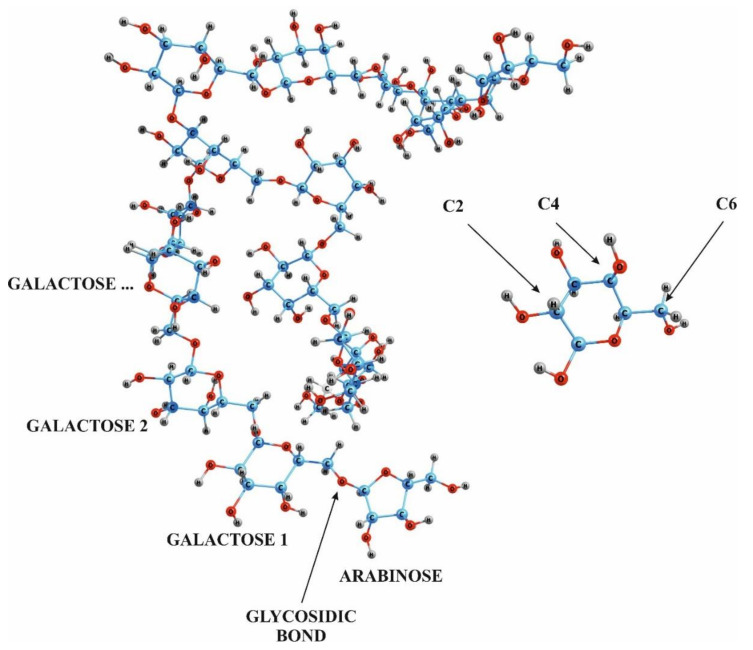
Fragment of the arabinogalactan molecule with marked hydroxyl groups responsible for the occurrence of predominant sulphation. The main chain consists of galactose units linked by glycosidic bonds, and the side chains consist of galactose and arabinose units and separate arabinose units.

It was found that in AG macromolecules from western larch, arabinose units are located at the ends of lateral branches consisting of three or four monosaccharide residues [27]. The content of glucuronic acid units in AG from various larch species is insignificant [27], and acid fragments were not found in purified AG samples from Western, European, Mountain, and Siberian larch. The macromolecule AG from larch wood has a highly branched structure; its main chain consists of galactose units linked by β-(1→3) glycosidic bonds, and side chains with β-(1→6) bonds- of galactose and arabinose units, of arabinose single units, and of uronic acids, mainly glucuronic acid. It is acknowledged that there are also arabinose units in the main macromolecule chain [29]. The ratio of galactose to arabinose units approximately is equal to 6:1, with one-third of the arabinose units in pyranose form, and two-thirds in furanose one [28,30]. It is expected that AG macromolecules probably exist in a very compact and spherical form (Figure 1) [32,33]

Arabinogalactan exhibits a wide range of biological properties: the immunobiological, hepatoprotective, antimutagenic, mitogenic, gastroprotective, and membranotropic activity; the probiotic, mycogenic, hypolipidemic, and immunomodulatory characteristics; the dispersing effect; etc. [27,29,30,31]. The macromolecule of arabinogalactan isolated from Siberian larch wood has a branched structure and a molecular weight of 15–20 kDa [28].

At the laboratory of natural synthons and ligands of the Irkutsk Institute of Chemistry, Siberian Branch, of the Russian Academy of Sciences, sulfated arabinogalactan in the form of a potassium salt was obtained by sulfation with a SO_3_-dimethylformamide complex in dimethyl sulfoxide [14]. The preclinical studies showed that the product is a promising lipid-lowering agent with the pronounced anticoagulant effect [14].

As is known, the anticoagulant activity of sulfated polysaccharides directly depends on the sulfation method used, which affects the degree of sulfation, arrangement of sulfate groups, molecular weight, etc. [3,15,16,17,18,19,20,21]. For example, drugs based on heparin, which are produced from lung tissue or mucose (intestinal mucosa) of animals, with subsequent purification and obtaining a heterogeneous substance with a molecular weight range from 3 to 30 kDa, with an average molecular weight of 15 kDa (15 to 100 monosaccharide residues). Such heparin in the current terminology is defined as unfractionated heparin (UFH) and only about 30% of UFH molecules have anticoagulant activity. A decrease in the molecular weight to 5.4 kDa (18–19 monosaccharide residues) causes significant qualitative changes in the activity of heparin. Low molecular weight heparins (LMWH) are produced by chemical or enzymatic depolymerization of unfractionated heparin (UFH) and are about one-third of the size of its molecule. LMWH consist of a mixture of polysaccharides with an average molecular weight of 4000–6000 Da [3,16,17,18,19,20,21].

Current methods of AG sulfates producing are based on the use of aggressive and environmentally hazardous sulfating agents such as sulfuric anhydride and chlorosulfonic acid [13,14,15,22,23,24,26]. We also developed a new, simple and environmentally friendly than known, method for the synthesis of sulfated arabinogalact by sulfation of arabinogalactan with sulfamic acid in dioxane in the presence of the main catalyst—urea [25]—and studied the physicochemical properties of the sulfated arabinogalactan obtained by this method [34]. Samples of sulfated arabinogalactan with different sulfur content were isolated depending on the process conditions. Therefore, controlling the characteristics of sulfated polysaccharides with a specified molecular weight as anticoagulants during their synthesis is of great practical importance in the continuation of these studies [25,34].

In the continuation of the work [25], the aims of this study were: (1) to study effect of urea-based activators on sulfation of arabinogalactan with sulfamic acid in 1,4-dioxane; (2) searching of the optimal parameters for obtaining arabinogalactan sulfate with the maximum sulfur content based on the obtained experimental data sulfating arabinogalactan with sulfamic acid in 1,4-dioxane in the presence of the main catalyst—urea and using the method of numerical optimization of the Box‒Behnken; (3) investigation of the dynamics of changes in molecular weight of sulfated arabinogalactan, obtained by sulfating arabinogalactan with sulfamic acid in 1,4-dioxane in the presence urea under different conditions by gel permeation chromatography for obtaining the sulfated product with the desired molecular weight characteristics.

## 2. Results and Discussion

### 2.1. Effect of Activators on Sulfation of Arabinogalactan with Sulfamic Acid in 1,4-Dioxane

**Figure 2:**
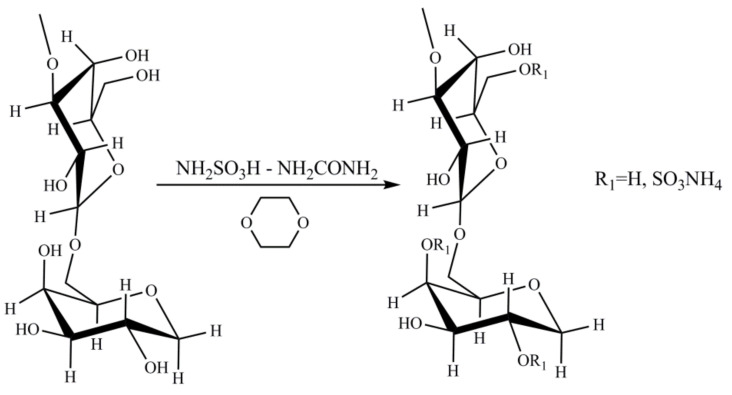
Scheme of sulfation of arabinogalactan.

As is known, the process of sulfation with sulfamic acid is activated by organic bases [35,36,37]. Sulfation of arabinogalactan with sulfamic acid in 1,4-dioxane in the presence of activators proceeds according to the scheme shown in Figure 2 [25].

In [37], the sulfation activators of different origins—including 1,4-dioxane,N,N-dimethylformamide, urea, pyridine, morpholine, and piperidine—were investigated. However, the effect of activators of the same origin on the process of sulfation with sulfamic acid has not been systematically studied. Here, we examine the effect of the urea-based activators on sulfation of arabinogalactan with sulfamic acid. The data are given in Table 1.

It was shown that, at the introduction of substituents into the urea molecule, their activating ability in the reaction of arabinogalactan sulfation with sulfamic acid decreases. In particular, the process activated by methylurea yields sulfated arabinogalactan with a sulfur content of 9.4 wt % and a M_w_ value of 12,310 Da. An increase in the substituent chain length in the urea molecule leads to a decrease in the sulfur content in arabinogalactan sulfate; for ethyl urea, it becomes 8.0 wt %. It should be noted that the use of the ethyl urea and hydroxyethyl urea activators ensures the same sulfur content (8.0 wt %) and PD value (1.22) in the product, with slightly different M_w_ values (10,969 and 11,130 Da, respectively). The use of biuret as an activator in the process of sulfation of arabinogalactan with sulfamic acid leads to the formation of a product with the lowest sulfur content (7.6 wt %).

The highest sulfur content (12.6 wt %) and molecular weight (19,667 Da) in arabinogalactan sulfated with sulfamic acid were obtained with the urea activator. The results are consistent with the literature data [37].

### 2.2. Numerical Optimization of the Process of Sulfation of Arabinogalactan with Sulfamic Acid in 1,4-Dioxane in the Presence of Urea

In this study, we exploited the effects of the process temperature and time and the amount of the sulfating complex on the sulfur content in the synthesized arabinogalactan sulfates.

The three factors included in the study as independent variables (the values are in parenthesis) were sulfation temperature X_1_ (75, 80, and 85 °C), sulfation time X_2_ (0.5, 1.75, and 3 h), and sulfating complex amount X_3_ per 1 g of arabinogalactan (10, 15, and 20 mmol). The output parameter of the sulfation process was sulfur content Y_1_ (wt %) in arabinogalactan sulfate. The Box‒Behnken experimental design (BBD) was used. Each experiment was carried out in duplicate. The designation of variables is given in Table 2.

The experimental results are shown in Table 3.

An increase in the process temperature should lead to an increase in the rate of sulfation, and the rate of depolymerization [38]. It is obvious that the low molecular weight fractions of arabinogalactan not only exhibit high reactivity in the sulfation reaction, but also undergo more rapid hydrolytic depolymerization under the action of sulfamic acid. Since the rate of depolymerization increases with process temperature [38], over time, a large amount of low molecular weight arabinogalactan sulfate with a sufficiently high sulfur content is formed, which is removed during dialysis purification (Section 2.3).

According to the data given in Table 3, the highest sulfur content in arabinogalactan sulfate (12.3 wt %) is obtained using 15 mmol of the sulfating complex at a process temperature of 85 °C and a process time of 3 h. The results of the variance analysis are given in Table 4.

The BBD experiment has proven useful in developing an accurate experimental model between important factors [39]. Experimental data on the sulfur content of arabinogalactan sulfates were analyzed by ANOVA. Significant factors were defined as *p* < 0.05. For all independent variables in the area of the factor space, in our case, *p* < 0.0043 was observed (Table 4).

The variance analysis showed that, under the established experimental conditions, the greatest contribution to the total variance of the output parameter is made by the temperature of the sulfation process. This is indicated by the high dispersion factors F for the main effects, also called the efficiencies (see Table 4).

The dependence of the sulfur content Y1 in arabinogalactan sulfates on the variable process factors is approximated by the regression equation:

Y1 = −111.536 + 2.27633X_1_ + 3.14067X_2_ + 1.0695X_3_ − 0.0103333X_1_^2^ + 0.004X_1_X_2_ − 0.013X_1_X_3_ − 0.437333X_2_^2^ −  0.064X_2_X_3_ + 0.00866667X_3_^2^ (1)

**Figure 3:**
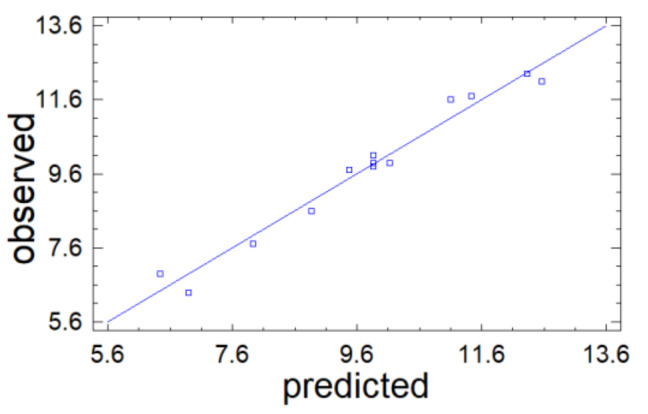
Results of observations vs. Y_1_ values predicted by mathematical model (1).

The predictive properties of Equation (1) are illustrated in Figure 3, in which the experimental output parameter Y_1_ is compared with its values calculated using Equation (1). The straight shows the calculated Y_1_ values and dots correspond to the results of observations. The proximity of the dots to the line confirms the good predictive properties of Equation (1).

**Figure 4:**
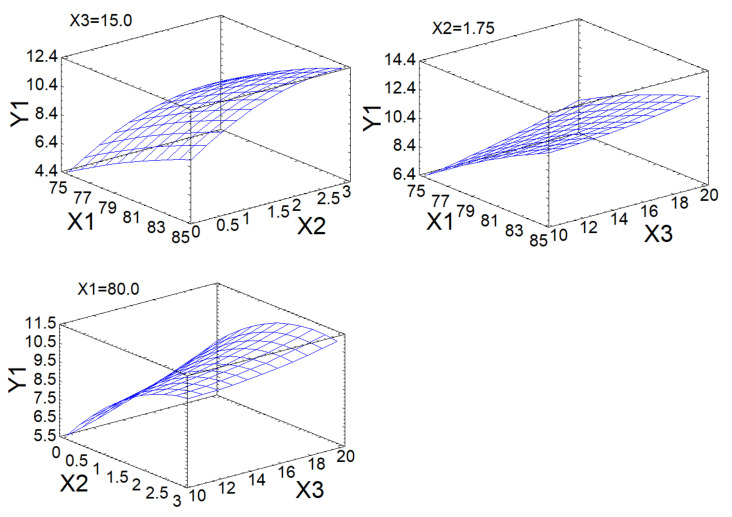
Response surface of the output parameters under different effects of the experimental conditions.

Figure 4 shows a graphical representation of Equation (1) in the form of a response surface.

The dependence of the output parameter Y1 on the variable factors X1 and X2 is described by a slightly curved response surface and reaches a ‘plateau’ after the X2 value of 2.5 h (Figure 3). The dependence of the output parameter Y1 on the variable factors X1 and X3 is described by an almost flat (with slight bends) response surface with a constant increase as the values of X1 and X3 increase. The dependence of the output parameter Y1 on the variable factors X2 and X3 has an ‘arched’ appearance with a maximum for X2 2 h, after which there is a slight decrease in values (Figure 4).

In addition, the quality of the approximation is characterized by the coefficient of determination R2adj. In the problem under consideration, it is R2adj = 94.0%, which indicates the high quality of the approximation. Therefore, we can state that the data obtained using Equation (1) are consistent with the observation results and this equation can be used as a mathematical model of the investigated process.

The calculated optimal parameters of the sulfation of arabinogalactan with sulfamic acid in dioxane in the presence of urea are a sulfamic acid amount of 15 mmol per 1 g of arabinogalactan, a temperature of 85 °C, and a time of 2.5 h.

### 2.3. Effect of the Method of Isolation of Sulfated Arabinogalactan on Its Molecular Weight Characteristics

**Figure 5:**
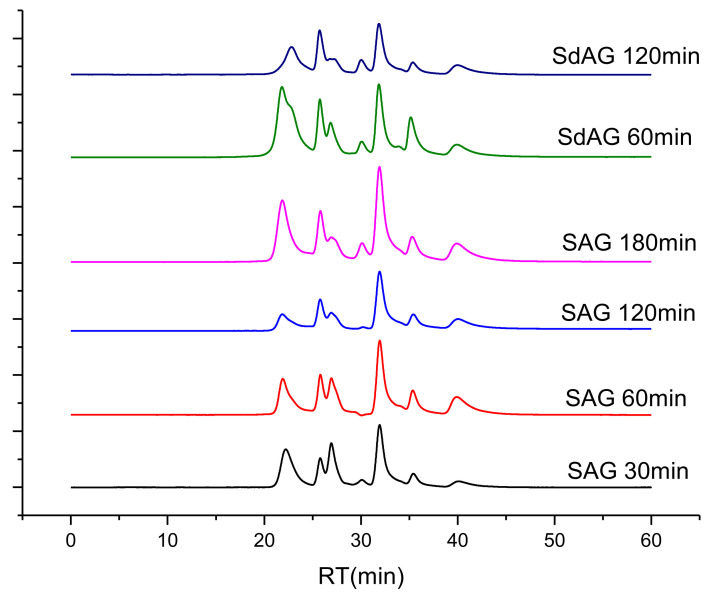
Gel permeation chromatograms of the solutions of sulfated arabinogalactan samples at a temperature of 85 °C, the ratio of the amount of the complex to the amount of the polysaccharide corresponds to 1:15 (g:mmol).

A classical method of purification of high molecular weight compounds from low molecular weight impurities is the dialysis process, in which low molecular weight components are removed through a semipermeable membrane. A membrane is conventionally a film based on cellulose derivatives with a pore size from 3 to 100 kDa. However, this polymer purification method is rather laborious. In view of this, we compared the molecular weight characteristics of the sulfated arabinogalactan samples obtained directly from the reaction medium and purified by dialysis. Chromatograms of the crude sulfated arabinogalactan samples are shown in Figure 5.

It was found that the main arabinogalactan peak with a retention time of ~22 min shifts to the left with an increase in the sulfation time, which indicates an increase in the mass of polymer chains due to the included sulfate groups. The peaks with a retention time of more than 25 min correspond to the initial reagents (sulfamic acid, urea, and dioxane) and to the low molecular weight reaction products.

The peaks corresponding to the main product are somewhat broadened, which can be attributed to the inhomogeneity of the initial arabinogalactan molecules and the fact that the low- and high-molecular chains are sulfated differently.

To remove the low molecular weight chains, the initial arabinogalactan was also dialyzed and then sulfated (Figure 5).

At a sulfation process time of 60 min, the ‘low molecular weight’ shoulder appears at the main arabinogalactan peak. As the time increases to 120 min, arabinogalactan completely passes to the low molecular weight form. In this case, the product yield was lower than 30%. These changes may indicate that, during dialysis of the initial arabinogalactan, the stabilizing polysaccharide chains were removed, which intensified the glycosidic bond breaking and the formation of low molecular weight products during sulfation.

**Figure 6:**
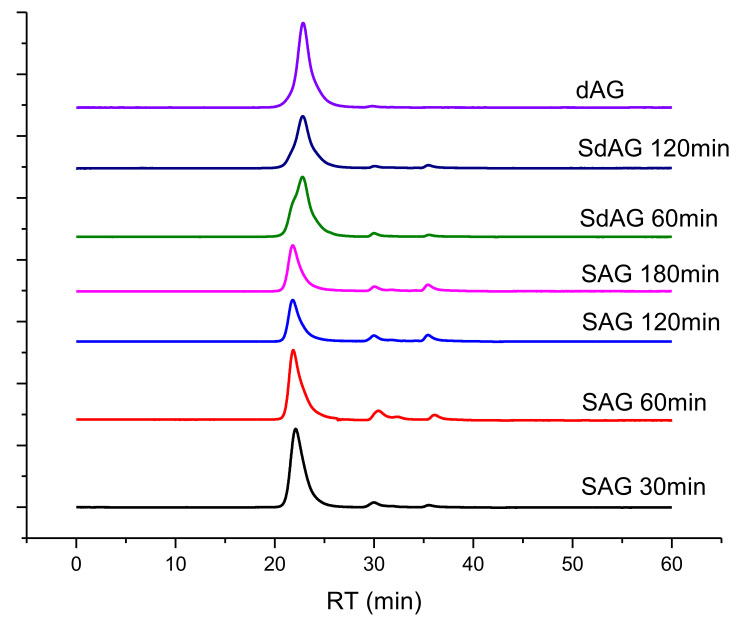
Gel permeation chromatograms of the sulfated arabinogalactan samples after dialysis (temperature of 85 °C and the ratio of the amount of the complex to the amount of the polysaccharide corresponds to 1:15).

The GPC data on the dialyzed sulfated arabinogalactan samples obtained at a temperature of 85 °C and the ratio of the amount of the complex to the amount of the polysaccharide corresponds to 1:15 (g:mmol) are presented in Figure 6.

According to the GPC data, during the dialysis, low molecular weight impurities were removed almost completely, while the retention time and form of the main product remained unchanged, in contrast to the case of the crude samples. Thus, the GPC technique can be used to analyze the molecular weight characteristics of crude products of sulfation of arabinogalactan without the additional laborious dialysis process. The use of cellophane bags in dialysis, which have smaller pore sizes than those used, could lead to the appearance of the low molecular weight fraction peak on the chromatogram and a different molecular weight distribution.

### 2.4. GPC Study of the Dynamics of the Molecular Weight Distribution of Sulfated Arabinogalactan

The GPC data on the MWD for the samples of the reaction mass of arabinogalactan sulfation at different process times and temperatures of 85 and 95 °C are given in Table 5 and Table 6, respectively.

The GPC data showed that, in contrast to the initial arabinogalactan, the number-average and weight-average molecular weights of sulfated arabinogalactan increase with the sulfation temperature already in the first 10–30 min of the process due to the introduction of sulfate groups; the degree of polydispersity changes insignificantly as compared with the value for the initial arabinogalactan and remains within 1.11–1.16 (Table 5).

Sulfation of arabinogalactan at a temperature of 85 °C and a process time of up to 90–120 min (Table 5) proceeds with an increase in the number-average and weight-average molecular weights of the sulfated polymer over the entire reaction time and is not accompanied by a significant change in the polydispersity of the reaction products, as compared with each other and with the initial arabinogalactan. The growth of the polymer molecular weight is apparently due to the change in the degree of sulfation of arabinogalactan, which increases with the process time [28]. The molecular weight characteristics of the investigated sulfated polymer suggest that, for a process time of up to 120 min, no significant destruction is observed in the initial and sulfated arabinogalactan. In 120 min, the molecular weights of the samples slightly decrease, which can be attributed to the onset of destruction processes. After dialysis of sulfated arabinogalactan at 85 °C for 180 min, the sample with a narrow MWD and a degree of polydispersity of 1.11 was obtained, which allows us to speak about purification from low molecular weight impurities and products of partial depolymerization of arabinogalactan.

The chromatographic study of the molecular weight characteristics of the arabinogalactan sulfation products obtained at 95 °C yielded different results (Table 6).

Up to a 50-min process time, the maximum peak coordinate M_p_ increases, most likely due to the introduction of sulfate groups into the macromolecule, while the M_w_ value decreases with an increase in polydispersity. Such a change in the molecular weight characteristics can result from the partial destruction of both the initial and sulfated arabinogalactan. To 120–180 min of the sulfation process, the molecular weights significantly decrease. A decrease in the molecular weight was previously observed during mechanochemical activation of arabinogalactan [29]. As was shown in [30], during sulfation of the mechanically activated arabinogalactan with a sulfuric anhydride‒pyridine complex in pyridine, with an increase in the sulfation temperature, the low molecular weight fraction in the sulfated product increases simultaneously with the sulfur content, which is due to the intensified partial destruction of the initial arabinogalactan and its sulfated derivatives.

The obtained results of the study of the molecular weight characteristics of the samples of the reaction mass sulfated at 85 and the duration of the process 40–180 min show that under these conditions the formation of sulfated products with close values of the average molecular weight, molecular weight distribution, and sulfur content (~12%). In contrast, the results of the chromatographic study of the molecular weight characteristics of the samples of the reaction mass sulfated at 95 °C and a process duration of 20–60 min show the possibility of obtaining sulfated arabinogalactan of different average molecular weight, but close molecular weight distribution and sulfur content (~12%). The results obtained in the future can be useful in studying the effect of the preparation conditions and the value of the molecular weight of the sulfated biopolymer on its anticoagulant activity.

## 3. Materials and Methods

### 3.1. Material

AG from the wood of the Siberian larch (*Larix sibirica Ledeb.*) produced by OOO “Khimiya drevesiny” (“Chemistry of wood”, Irkutsk, Russia) under the trade name “FibrolarS” was used as the initial material.

### 3.2. Synthesis of Sulfated Arabinogalactan

Sulfation of arabinogalactan with sulfamic acid in 1,4-dioxane in the presence of urea, ethylurea, biuret, methylurea and hydroxyethyl urea (base), using the modified procedure, described in [28]. 1,4-dioxane (50 mL), sulfamic acid 4.9–9.7 g (50–100 mmol), and suitable base catalyst (50–100 mmol) were placed in a three-necked bottle equipped with a thermometer, a mechanical stirrer, and a water bath, the mixture was heated under intensive stirring to a fixed value (according to the sulfation conditions given in Table 1 and Table 3), air-dried AG (5 g) was added, and the mixture was stirred at this temperature for 0.5–3 h. After the termination of sulfation, the solvent was decanted, the resulting viscous residue was dissolved in water (25 mL), and excessive sulfamic acid was neutralized by a 25% aqueous ammonia solution to the neutral reaction and poured into ethanol (100 mL). The resulting viscous product was separated and washed three times by ethanol (10 mL each portion) until a solid precipitate formed. The precipitate, which was a sulfated AG derivative in the form of ammonium salt, was washed on a filter with 10 mL of ethanol and dried in air. The ammonium salt of sulfated AG was dissolved in 30 mL of distilled water and was purified by the dialysis on cellophane against distilled water. The product was dialyzed for 10–15 h; water was changed at 1–2 h intervals. Dialysis bag MF-5030-46 (MFPI, Seguin, TX, USA) with a pore size of 3.5 kDa and width of 46 mm was used. For the comparison of the molecular weight characteristics of the sulfated arabinogalactan obtained at different temperatures and duration of the sulfation process, a portion of the reaction mass was taken and was studied by gel permeation chromatography (Figure 4 and Figure 5; Table 5 and Table 6).

### 3.3. Experimental Methods

The dynamics of the molecular weight characteristics of the reaction mass during the sulfation of arabinogalactan was studied by gel permeation chromatography (GPC). The reaction mass samples (100–200 mg) for the GPC investigations were taken after 10, 20, 30, 40, 50, 60, 90, 120, 150, and 180 min of the process, dissolved in 5 mL of distilled water, and neutralized with an aqueous solution of ammonia to pH 7.

The molecular weight distribution (MWD) of the reaction mass samples was determined on an Agilent 1200 Infinity II multi-detector GPC/SEC system with triple detection: refractometer (PL), viscometer (VS) and light scattering (LS). The separation was made on two Aquagel-OH Mixed-M columns (300 × 7.5 mm, 8 µm) using the aqueous solution of 0.1 M NaNO_3_ (pH = 7) as a mobile phase. The column was calibrated by the polyethylene glycol standards (Agilent, Santa Clara, CA, USA). The eluent flow rate was 1 mL/min and the sample volume was 100 μL. Before the analysis, the samples were dissolved in the mobile phase (1 mg/mL) and filtered through a 0.22 μm hydrophilic PTFE membrane filter (Agilent). Data collection and data processing were performed using the Agilent GPC/SEC MDS software.

The sulfur content was determined on a Thermo Quest Flash EA-1112 thermal analyzer (Thermo Electron, Milan, Italy). This section may be divided by subheadings. It should provide a concise and precise description of the experimental results, their interpretation, as well as the experimental conclusions that can be drawn.

The software Statgraphics Centurion XVI V16.1.09, DOE block (design of experiment), was used for numerical optimization of the AG sulfation process [40].

## 4. Conclusions

In this study, the dynamics of the molecular weight characteristics of sulfated arabinogalactan upon variation in the temperature and time of sulfation of arabinogalactan with sulfamic acid in 1,4-dioxane was investigated.

It was found that, during sulfation of arabinogalactan at 85 °C with an increase in the process time from 10 to 90 min, the molecular weights of the reaction products increase due to the introduction of sulfate groups without significant destruction of the initial polymer and sulfation products. Sulfation at 95 °C for 20 min leads to the formation of the products with a higher molecular weight than at 85 °C, since the sulfation rate increases; however, in the further course of the process under these conditions, sulfation is accompanied by destruction and the molecular weight of the sulfated polymer decreases.

The conditions for obtaining sulfated arabinogalactan with the desired molecular weight characteristics were established.

The effect of urea-based activators on sulfation of arabinogalactan with sulfamic acid was examined. It was shown that an increase in the substituent chain length leads to a decrease in the sulfur content in arabinogalactan sulfate. It was found that urea exhibits the best activating ability among the investigated activators.

The Box‒Behnken numerical optimization of the process of sulfation of arabinogalactan with sulfamic acid in 1,4-dioxane was performed. It was shown that the optimal parameters for obtaining arabinogalactan sulfate with the maximum sulfur content (12.8 wt %) are a sulfamic acid amount of 20 mmol per 1 g of arabinogalactan, a process temperature of 85 °C, and a process time of 2.5 h.

## Figures and Tables

**Table 1:** Effect of the urea-based activators on the MWD and sulfur content in arabinogalactan sulfated with sulfamic acid in 1,4-dioxane at 85 °C for 3 h, the ratio of the amount of the complex to the amount of the polysaccharide corresponds to 1:15 (g:mmol).

No.	Sample	Activator	Activator Formula	Mp	M_n_ (Da)	M_w_ (Da)	PD	Sulfur Content, wt %
1	Initial arabinogalactan	-	-	9451	7998	10,340	1.29	
2	Sulfated arabinogalactan	urea	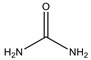	19,902	17,661	19,667	1.11	12.6
3	Sulfated arabinogalactan	ethyl urea	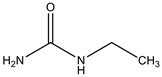	9838	8968	10,969	1.22	8.0
4	Sulfated arabinogalactan	biuret	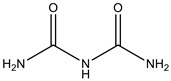	9681	8591	10,953	1.27	7.6
5	Sulfated arabinogalactan	hydroxyethyl urea	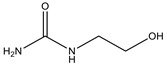	9838	9155	11,130	1.22	8.0
6	Sulfated arabinogalactan	methyl urea	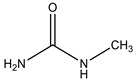	10,406	9567	12,310	1.29	9.4

**Table 2:** Independent factors and output parameter (experimental results).

Factors and Parameters	Designation in the Equations	Range
Process temperature, °C	X1	75–85
Process time, h	X2	0.5–3.0
Amount of sulfating complex per 1 g AG, mmol	X3	10–20
Sulfur content, wt %	Y1	-

**Table 3:** Effect of the conditions for arabinogalactan sulfation with sulfamic acid in 1,4-dioxane in the presence of urea on the degree of substitution in arabinogalactan sulfates.

№	Temperature, °C	Process Time, h	Amount of Sulfating Complex Per 1 g AG, mmol	Sulfur Content, wt %
1	80	1.75	15	9.9
2	75	0.5	15	5.6
3	85	0.5	15	10.1
4	75	3	15	7.7
5	85	3	15	12.3
6	75	1.75	10	6.9
7	85	1.75	10	11.7
8	80	1.75	15	9.8
9	75	1.75	20	8.6
10	85	1.75	20	12.1
11	80	0.5	10	6.4
12	80	3	10	9.9
13	80	0.5	20	9.7
14	80	3	20	11.6
15	80	1.75	15	9.9

**Table 4:** Result of the variance analysis.

Variance Source	Statistical Characteristic	
	*F* Factor	*p* Value
X1	146.78	0.0001
X2	45.62	0.0011
X3	24.44	0.0043
X12	0.96	0.3732
X1X2	0.01	0.9254
X1X3	1.64	0.2567
X22	6.69	0.0491
X2X3	2.48	0.176
X32	0.67	0.4496
Df	14	
R2	97.9	
R2adj	94.0	

**Table 5:** MWD for the samples of the reaction mass sulfated at a temperature of 85 °C, the ratio of the amount of the complex to the amount of the polysaccharide corresponds to 1:15.

Sample No.	Time, min	M_p_, Da	M_n_, Da	M_w_, Da	M_n_/M_w_	%S
AG	‒	15,601	14,393	16,789	1.16	
1	10	17,111	15,181	17,525	1.15	7.79
2	20	18,369	15,492	17,418	1.12	9.82
3	30	19,122	16,117	18,032	1.12	11.047
4	40	19,902	16,776	18,756	1.12	12.30
5	50	19,902	16,772	18,786	1.12	12.30
6	60	20,101	16,941	18,915	1.12	12.62
7	90	20,302	17,177	19,084	1.11	12.95
8	120	20,101	16,979	18,930	1.12	12.62
9	150	19,908	16,737	18,740	1.12	12.31
10	180	19,903	16,689	18,692	1.12	12.30
11 *	180	19,902	17,661	19,667	1.11	12.30

* Dialysis-purified sulfated arabinogalactan (the sulfur content is 12.3 wt %).

**Table 6:** MWD for the samples of the reaction mass sulfated at a temperature of 95 °C, the ratio of the amount of the complex to the amount of the polysaccharide corresponds to 1:15.

Sample No.	Time, min	M_p_, Da	M_n_, Da	M_w_, Da	Degree of Polydispersity, M_n_/M_w_	%S
AG	‒	15,601	14,393	16,789	1.16	
1	10	20,101	16,850	18,803	1.12	12.62
2	20	20,915	16,221	18,331	1.13	13.93
3	30	20,709	15,241	17,343	1.14	13.60
4	40	20,504	15,019	17,249	1.14	13.27
5	50	20,504	14,362	16,660	1.16	13.27
6	60	20,302	12,939	15,010	1.16	12.94
7	90	18,184	12,755	14,924	1.17	9.52
8	120	16,857	12,236	14,439	1.18	7.37
9	150	14,504	12,206	14,282	1.17	3.57
10	180	13,905	11,879	13,889	1.17	2.60
11 *	180	16,937	8440	12,661	1.50	13.9

* Dialysis-purified sulfated arabinogalactan (the sulfur content is 13.9 wt %).

## Data Availability

All data generated during this study are included in this article.

## Sample Availability

Samples of the compounds are not available from the authors.
